# The anti-oxidative transcription factor Nuclear factor E2 related factor-2 (Nrf2) counteracts TGF-β1 mediated growth inhibition of pancreatic ductal epithelial cells -Nrf2 as determinant of pro-tumorigenic functions of TGF-β1

**DOI:** 10.1186/s12885-016-2191-7

**Published:** 2016-02-25

**Authors:** Geeske Genrich, Marcus Kruppa, Lennart Lenk, Ole Helm, Anna Broich, Sandra Freitag-Wolf, Christoph Röcken, Bence Sipos, Heiner Schäfer, Susanne Sebens

**Affiliations:** Group Inflammatory Carcinogenesis, Institute for Experimental Cancer Research, Christian-Albrechts-University Kiel, Arnold-Heller-Str. 3, Building 17, 24105 Kiel, Germany; Institute of Medical Informatics and Statistics, UKSH Campus Kiel, Brunswiker Str. 10, 24105 Kiel, Germany; Department of Pathology, Christian-Albrechts-University Kiel, Arnold-Heller-Str. 3, Building 14, 24105 Kiel, Germany; Department of Pathology and Neuropathology, University Hospital Tübingen, Liebermeisterstr. 8, 72076 Tübingen, Germany; Laboratory of Molecular Gastroenterology & Hepatology, Institute for Experimental Cancer Research, Christian-Albrechts-University Kiel, Arnold-Heller-Str. 3, Building 6, 24105 Kiel, Germany

**Keywords:** Chronic pancreatitis, Pancreatic cancer precursor lesion, Oxidative stress, TGF-β1, Proliferation, Pancreatic cancer

## Abstract

**Background:**

Nuclear factor E2 related factor-2 (Nrf2) is an oxidative stress inducible transcription factor being essential in regulating cell homeostasis. Thus, acute induction of Nrf2 in epithelial cells exposed to inflammation confers protection from oxidative cell damage and mutagenesis supporting an anti-tumorigenic role for Nrf2. However, pancreatic ductal adenocarcinoma (PDAC) is characterized by persistent Nrf2 activity conferring therapy resistance which points to a pro-tumorigenic role of Nrf2. A similar dichotomous role in tumorigenesis is described for the Transforming Growth Factor-beta 1 (TGF-β1). The present study therefore aimed at elucidating whether the switch of Nrf2 function towards a tumor promoting one relates to the modulation of TGF-β1 induced cell responses and whether this might occur early in PDAC development.

**Methods:**

*In situ* analysis comprised immunohistochemical stainings of activated (phosphorylated) Nrf2 and Ki67 in pancreatic tissues containing normal ducts and pancreatic intraepithelial neoplasia (PanINs). *In vitro*, Nrf2 levels in benign (H6c7-pBp), premalignant (H6c7-kras) and malignant (Colo357) pancreatic ductal epithelial cells were modulated by Nrf2 specific siRNA or Nrf2 overexpression. Then, the effect of Nrf2 alone and in combination with TGF-β1 on cell growth and survival was investigated by cell counting, Ki67 staining and apoptosis assays. The underlying cell signaling was investigated by western blotting. Statistical analysis was performed by Shapiro-Wilk test for normal distribution. Parametric data were analyzed by one-way ANOVA, while non-parametric data were analyzed by Kruskal-Wallis one-way ANOVA on ranks.

**Results:**

Significantly elevated expression of activated Nrf2 and Ki67 could be detected in PanINs but not in normal pancreatic ductal epithelium. While the effect of Nrf2 on basal cell growth of H6c7-pBp, H6c7-kras and Colo357 cells was minor, it clearly attenuated the growth inhibiting effects of TGF-β1 in all cell lines. This enhanced Nrf2-mediated cell survival was predominantly based on an enhanced proliferative activity. Accordingly, expression of p21 expression along with expression of phospho-p38 and phospho-Smad3 was diminished whereas Erk-phosphorylation was enhanced under these conditions.

**Conclusions:**

Overall, our data demonstrate that Nrf2 being elevated in early precursor lesions counteracts the growth inhibiting function of TGF-β1 already in benign and premalignant pancreatic ductal epithelial cells. This could represent one fundamental mechanism underlying the functional switch of both- TGF-β1 and Nrf2 – which may manifest already in early stages of PDAC development.

**Electronic supplementary material:**

The online version of this article (doi:10.1186/s12885-016-2191-7) contains supplementary material, which is available to authorized users.

## Background

Pancreatic ductal adenocarcinoma (PDAC) is still a leading cause of cancer related deaths in western countries with a poor 5-year survival rate of 6 % [1]. This can be mainly explained by the late diagnosis when the disease has been already progressed to an advanced stage and a profound therapy resistance. Accordingly, much effort is given to a better understanding of the early steps of PDAC development in order to identify targets that can be used for screening tests, early diagnosis and/or chemoprevention.

Precursor lesions of PDAC predominantly originate from ductal cells with pancreatic intraepithelial neoplasia (PanIN) being the most frequent and best characterized precursor lesions of PDAC [2]. One of the earliest genetic alterations which is present in 99 % of even early PanINs (PanIN1) is the mutation of the oncogene *kras* [3] being an essential driver of PDAC development [4]. Besides the genetic alterations, one hallmark of PDAC is its pronounced stromal microenvironment comprising stellate cells, myofibroblasts and diverse immune cells together with extracellular matrix [5–8] which start to accumulate in earliest PanINs [9].

Besides cancer cells themselves, myofibroblasts and immune cells such as macrophages are a main source of Transforming Growth Factor-beta1 (TGF-β1) [5, 10]. Although TGF-β1 is able to potently inhibit the growth of cells including transformed cells and thereby acts as tumor suppressor, it also represents an important key driver in tumor development, e.g. of PDAC, by promoting invasion, metastasis and chemoresistance of tumor cells as well as immunosuppression and angiogenesis [10, 11]. TGF-β1 can exert its pleiotropic functions via the Smad-dependent (canonical) signaling pathway or via signaling through various Smad-independent pathways e.g. the Mitogen-activated protein kinases (MAPK) p38 and Erk1/2, the latter ones contributing to TGF-β1 responsiveness even in the presence of mutations in the *Smad4/DPC4* gene [12]. Thus, the function of TGF-β1 is a double-edged sword and the switch from tumor suppressor to a tumor promoter seems to be context dependent, albeit the exact underlying mechanisms are still poorly understood [12, 13].

A similar dual role in tumorigenesis has been described for the antioxidative transcription factor Nuclear factor E2 related factor-2 (Nrf2) [14–16]. In response to metabolic, xenobiotic or oxidative stress (e.g. in the course of inflammation), Nrf2 becomes activated leading to transcription of a variety of genes contributing to restoration of redox and cell homeostasis, e.g. antioxidant enzymes NAD(P)H dehydrogenase [quinone] 1 (NQO1), Hemoxygenase (HO)-1, anti-apoptotic proteins such as Bcl-2 or metabolic enzymes [16]. However, constitutive high expression and activity of Nrf2 have been described for several tumors including PDAC [17] contributing to chemo-/radioresistance [18–21], enhanced cell motility [22], metabolic reprogramming [23], maintenance of self-renewal of cancer stem cells [24] as well as enhanced proliferation [17, 25]. In an endogenous PDAC mouse model it was shown that oncogenic *kras* signaling leads to tumor cell proliferation and tumorigenesis via elevation of Nrf2 activity [25]. The fact that TGF-β1 and Nrf2 both become upregulated upon persistent inflammation suggests that these two factors may virtually impact on their signaling pathways paving the way for their switch from tumor suppressor to tumor promoter. Accordingly, it has been shown that Nrf2 can inhibit the profibrotic action of TGF-β1 by preventing Smad3 activation [26, 27]. Thus, the present study intends to investigate whether Nrf2 contributes to the pro-tumorigenic switch of TGF-β1 in PDAC by antagonizing the TGF-β1 mediated growth inhibiting effect on pancreatic ductal epithelial cells thereby undergoing a functional switch itself. In order to verify whether this switch might occur at early stages of PDAC development, particular emphasis was given to the *in situ* analyses of activated Nrf2 and Ki67 in early PanINs. *In vitro*, the interplay of Nrf2 and TGF-β1 on cell growth was investigated on three pancreatic ductal epithelial cell lines resembling different stages of PDAC development, namely benign H6c7-pBp, premalignant H6c7-kras and malignant Colo357 cells. Overall, our study provides ample evidence that the functional switch of Nrf2 occurs early in PDAC development based on its ability to counteract the growth inhibiting function of TGF-β1.

## Methods

### Cell lines and cell culture

As model for benign pancreatic ductal epithelium, the human pancreatic ductal epithelial cell line H6c7-pBp and as model for premalignant pancreatic ductal epithelium harboring a kras^G12V^ mutation, the cell line H6c7-kras [28] were used, both well-established cell models and kindly provided by M.S. Tsao (Ontario Cancer Institute, Toronto, Canada). Both cell lines were cultured in H6c7-medium (50 % RPMI 1640 medium (Biochrom, Berlin, Germany) and 50 % KSF-medium (Gibco Life Technologies, Darmstadt, Germany) supplemented with 5 % fetal calf serum, 0.5 % L-glutamine (both Biochrom), 50 μg/mL bovine pituitary extract, 5 ng/mL epidermal growth factor (both Gibco Life Technologies) + 0.5 μg/mL puromycin (Invitrogen, Darmstadt, Germany). The human pancreatic ductal epithelial cell line Colo357 was kindly provided by H. Kalthoff, Institute of Experimental Cancer Research, Kiel, Germany) and kept in Colo357-medium which is composed of RPMI-1640 medium supplemented with 1 % L-glutamine, 10 % FCS and 1 % sodium pyruvate. Colo357 cells used in this study harbor a wild type Smad4/DPC4 genotype [29] and are genetically distinct from those Colo357 cells having a homozygous deletion of the Smad4 gene [30].

### Knock-down of Nrf2

To suppress endogenous Nrf2 expression, 1 × 10^5^ cells/well of H6c7-pBp, H6c7-kras and Colo357 cells were seeded in 12-well plates containing 1 mL medium/well. For transfection, medium was removed and 1 mL fresh medium was added. Then, 6 μL/well HiperFect reagent (Qiagen, Hilden, Germany) and 75 ng/well of either negative control siRNA or specific Nrf2 siRNA (no. SI03246614, both from Qiagen) were mixed with 100 μL FCS-free medium and added to the cells. Specificity of the siRNA was confirmed previously [18]. After 24 h, cells were either left untreated or treated with 10 ng/mL TGF-β1 (BioLegend, Fell, Germany) for 48 h.

### Overexpression of Nrf2

To overexpress Nrf2 in H6c7-pBp and H6c7-kras cells, either cell line was seeded into 6 well plates (2 x 10^5^ cells/well). After 24 h, medium was removed and replaced by 1,6 mL fresh medium. Then, 100 μL/well EC-puffer, 8 μL/μg plasmid Enhancer and 20 μL/μg plasmid Effectene (all from Qiagen) together with 0,3 μg/well of either pcDNA3.1 control vector (pcDNA3.1; Invitrogen) or pcDNA3.1 encoding Nrf2-HA were mixed and added to the cells. After 16 h, medium was replaced by 2 mL/well of fresh H6c7- or Colo357 medium and cells were either left untreated or treated with 10 ng/mL TGF-β1 for 48 h.

### Western blotting

Preparation of whole cell lysates and nuclear extracts as well as electrophoresis and western blotting have been described elsewhere [31, 32]. The following antibodies were used according to the manufacturer’s instructions: rabbit anti-HSP90α/β (clone H-114), mouse anti-lamin-A/C (clone 346), goat anti-Smad2/3 (clone E-20) (all from Santa Cruz, Heidelberg, Germany), rabbit anti-Erk1/Erk2, rabbit anti-phospho Erk1/Erk2 (T204/Y209), rabbit anti-p38, mouse anti-phospho p38 (T180/Y182, clone D3F9), rabbit anti-phospho Smad3 (Ser423/425), rabbit anti-PARP (all from Cell Signaling via New England Biolabs, Frankfurt/a.M., Germany), mouse anti-p21 (clone 187) (BD Biosciences, Heidelberg, Germany), monoclonal rabbit anti-Nrf2 (clone EP1808Y, Abcam, Berlin, Germany), mouse anti-tubulin (clone B-5-1-2) and rabbit anti-HA (both from Sigma-Aldrich, Taufkirchen, Germany). Primary antibodies were incubated overnight at 4 ° C and detected by anti-rabbit, anti-goat or anti-mouse HRP-linked antibodies (Cell Signaling) at room temperature for 1 h. After washing in TBST, blots were developed with SuperSignal West Dura Extended Duration Substrate (Perbio Sciences, Bonn, Germany). Average band intensities were determined by densitometry using ImageI 1.47v software (National Institute of Health). Values of the proteins of interest were divided by the values of the corresponding loading control (Hsp90). Additionally, values of phosphorylated proteins were divided by the values of the corresponding total protein (data are presented in Additional file 1: Figure S2A + C).

### Determination of vital cell number

After detachment with trypsin-EDTA (PAA, Pasching, Austria), cells were stained with trypan blue (Sigma-Aldrich, Munich, Germany) and counted using a Neubauer counting chamber. For quantification of vital cells, blue stained cells were excluded from counting.

### Ki67 staining

For Ki67 staining, cells were seeded on cover slips (ThermoScientific, Schwerte, Germany) before transfection and stimulation procedures. Then, medium was removed, cells were washed with PBS and fixed with ice-cold acetone + 0.3 % H_2_O_2_ for 10 min. After washing in PBS, cells were blocked in 4 % BSA/PBS for 20 min. Cells were incubated with 2 μg/mL mouse IgG1 anti-Ki67 antibody (BD Biosciences, Heidelberg, Germany) diluted in 1 % BSA/PBS at RT for 45 min. Cells were washed with PBS and incubated with EnVision-HRP anti-mouse (Dako, Hamburg, Germany) for 30 min, washed with PBS and incubated with AEC Substrate (Dako) for 2-10 min. After final washing in PBS, cells were stained in Mayer’s Haemalaun (AppliChem, Darmstadt, Germany) for 2 min. After washing in water for 10 min, cover slips were fixed with Kaiser’s glycerine gelatine (Waldeck, Münster, Germany). Initial isotype control stainings were performed with a mouse IgG1 antibody (R&D Systems, Wiesbaden, Germany) under identical conditions revealing no staining. Evaluation was done using an Evos_xL_ Core microscope (AMG, Bothell, USA). Quantification of Ki67 positive cells was performed at a 200-fold magnification by choosing 5 representative visual fields in each counting positively stained as well as negative cells along a diagonal line using Microsoft Powerpoint 2007 in order to calculate the percentage of Ki67 positive cells. If less than ten cells touched the line, all captured cells of the visual field were counted.

### Measurement of apoptosis

Determination of caspase-3/7 activity was performed in transfected and stimulated H6c7-kras and Colo357 cells using a Caspase-Glo® 3/7 assay (Promega, Mannheim, Germany), according to the manufacturer’s instructions and as described [32, 33]. All samples were measured in duplicates.

### Immunohistochemistry

Paraffin-embedded and formalin-fixed postmortem pancreatic tissues of 22 individuals that had died of non-pancreatic diseases were used for immunohistochemical analysis. The research was approved by the ethics committee of the Semmelweis University, Budapest, Hungary (140-1/1996). The need for an informed consent was waived by the ethics committee of the Semmelweis University, Budapest, Hungary according to national regulations. Only tissues that have been extensively characterized were used [34, 35]. Consecutive 3 μm thick tissue sections were deparaffinized and rehydrated as previously described [8]. Staining for Ki67 (clone SP6, Fisher/Thermo Scientific) was performed at the Institute of Pathology using an automated routine procedure. For staining of p-Nrf2, antigen retrieval was performed with 1:10 diluted citrate buffer at pH 6.0 for 20 min. After washing, sections were incubated either with a monoclonal rabbit anti-phospho(Ser40)-Nrf2 (1 μg/ml; clone EP18094, Abcam) diluted in 1 % BSA and 0.3 % Triton-X-100/PBS at 4 °C overnight. After washing, sections were incubated with EnVision-HRP anti-rabbit (Dako) for 45 min at room temperature. Substrate reaction was performed with AEC Substrate (Dako) for 10 min. After washing, cells were stained in Mayer’s Haemalaun (Merck, Darmstadt, Germany) for 2 min. After washing in water for 10 min, sections were covered with Kaiser’s glycerine gelatine (Roth, Karlsruhe, Germany). Usage of control antibodies revealed no or only weak background staining.

### Evaluation

Stained tissue sections were evaluated twice in a blinded manner by scoring the extent of distribution (given as %-positivity of the whole section). In case of two discrepant results, sections were evaluated by a second investigator. If possible, 10 normal ducts and PanINs were analysed in each pancreatic tissue. Each parameter was evaluated using a 5-score system (1 = negative, 2 = <10 % positive, 3 = 11–50 % positive, 4 = 51–90 % positive, 5 = more than 90 % positive). To enable performance of statistical analysis, the created groups of tissue samples per score were then dichotomized by the median score and refitted into two groups of < median (low expression) and ≥ median (high expression). Evaluation of the sections was carried out using an Axioplan 2.0 microscope (Zeiss, Jena, Germany). Pictures were taken using a Keyence BZ9000 microscope (Keyence, Neu-Isenburg, Germany).

### Statistical analysis

Relationships between data from immunohistochemical stainings of pancreatic tissues were categorized and compared between groups by Fisher’s exact test using SPSS 17.0 (IBM, Ehningen, Germany). Statistical analysis of *in vitro* data was performed using SigmaPlot Software 12.5 (Systat Software GmbH, Erkrath, Germany). The Shapiro-Wilk test was used to test for normal distribution. Parametric data were analyzed by one-way RM ANOVA, while non-parametric data were analyzed by Kruskal-Wallis one-way ANOVA on ranks test. P-values < 0.05 were regarded as statistically significant and are indicated with an asterisk (*).

## Results

### Expression of activated Nrf2 is elevated in early PanINs correlating with an increased proliferative activity

**Fig. 1:**
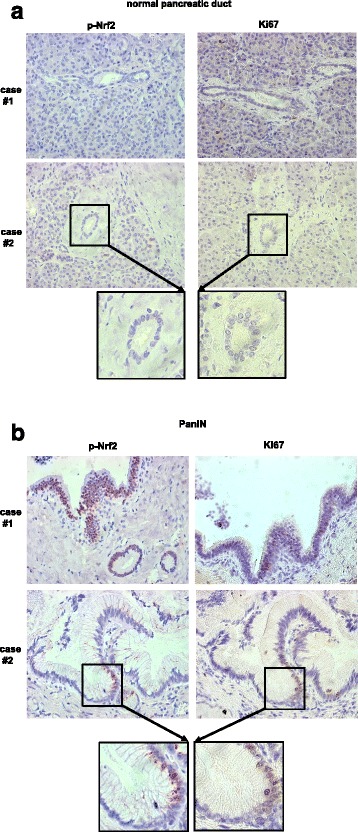
Expression of activated Nrf2 is elevated in PanINs correlating with an increased proliferative activity. Representative immunhistochemical stainings of activated (phosphorylated) Nrf2 (p-Nrf2) and Ki67 in (**a**) normal pancreatic ducts and (**b**) PanINs of two individuals. Magnification x400 and x800 (frames)

To elucidate whether early PanIN lesions already exhibit greater Nrf2 activity and whether this correlates with an increased proliferative activity, phospho-Nrf2 (p-Nrf2), representing activated Nrf2, as well as Ki67 were immunohistochemically detected and scored in normal ducts and PanINs of pancreatic tissues from 22 individuals which had died from non-pancreatic diseases (Tables 1 and 2). While the majority of normal ducts showed no or only weak p-Nrf2 expression (16/21 with low expression, Fig. 1a), significantly elevated p-Nrf2 expression (= high expression) was detected in PanINs of 17/20 tissues (Fig. 1b). Ki67 expression was elevated in 45 % (10/22 with high expression) of normal ducts, but in all analyzed PanINs (Fig. 1, Tables 1 and 2). Moreover, this elevated proliferative activity of PanINs was significantly associated with increased p-Nrf2 expression (Table 3) supporting the view that Nrf2 activity accounts for higher proliferation of pancreatic ductal epithelial cells already in early PanINs.

**Table 1:** Initial scoring of p-Nrf2 and Ki67 expression in normal pancreatic ducts and PanINs

	Normal ducts					PanINs					
Marker	Score 1	Score 2	Score 3	Score 4	Score 5	Score 1	Score 2	Score 3	Score 4	Score 5	Median score
p-Nrf2	16	5	0	0	0	3	13	4	0	0	2
Ki67	12	10	0	0	0	0	15	4	0	0	2

**Table 2:** Interrelationship of p-Nrf2 and Ki67 expression in normal pancreatic ducts and PanINs, initial scores were dichotomized by the median score and refitted into 2 groups of low and high expression

	Normal ducts (*n* = 22)		PanINs (*n* = 20)		*p*-value
Marker	Low	High	Low	High	
p-Nrf2	16/21	5/21	3/20	17/20	0.002
Ki67	12/22	10/22	0/19	19/19	0.001

In two cases, no PanINs were detectable

**Table 3:**
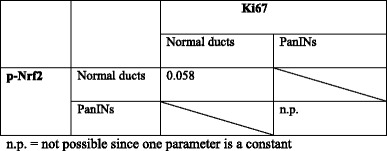
Association between p-Nrf2 and Ki67 expression in normal pancreatic ducts and PanINs, expressed as p-values from chi-square test

### Nrf2 antagonizes the growth inhibiting effect of TGF-β1 on benign, premalignant and malignant pancreatic ductal epithelial cells

**Fig. 2:**
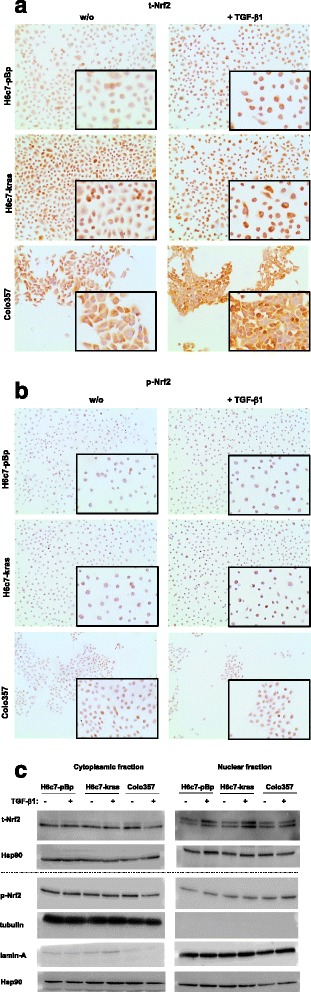
Different Nrf2 expression in benign, premalignant and malignant pancreatic ductal epithelial cells. H6c7-pBp, H6c7-kras and Colo357 cells were either left untreated or were treated with 10 ng/ml TGF-β1 for 48 h. Representative immunocytochemical stainings of (**a**) total (t)-Nrf2 and (**b**) phospho (p)-Nrf2. Magnification x 280 (magnification of images in frames x 540). **c** Representative western blots showing t-Nrf2 and p-Nrf2 expression in nuclear extracts of H6c7-pBp, H6c7-kras and Colo357 cells either left untreated or treated with 10 ng/ml TGF-β1 for 48 h. Lamin-A, tubulin and Hsp90 were detected as loading control for nuclear, cytosolic and total cell extracts, respectively

Animal based studies revealed that oncogene activation can lead to enhanced Nrf2 mediated gene transcription accounting for increased proliferation and tumorigenesis [25]. To investigate the impact of Nrf2 on cell growth of human pancreatic ductal epithelial cells, pancreatic ductal epithelial cell lines were used resembling different stages of PDAC development: 1) benign H6c7-pBp cells lacking any of the known mutations driving pancreatic tumorigenesis [28], 2) premalignant H6c7-kras cells harboring a kras^G12V^ mutation [28] and 3) malignant Colo357 cells [36]. Since TGF-β1 is elevated at early stages of PDAC development, too, the investigations focused on the interplay between Nrf2 and TGF-β1. As demonstrated by immuncytochemical staining (Fig. 2a,b) and western blotting (Fig. 2c), basal nuclear Nrf2 expression could be detected in all three pancreatic duct cell lines with the lowest expression in benign H6c7-pBp cells and the highest nuclear Nrf2 expression in malignant Colo357 cells (Fig. 2a-c). In line with our recent findings [37], treatment with 10 ng/ml TGF-β1 for 48 h led to an induction of Nrf2 expression in all three cell lines (Fig. 2a-c).

**Fig. 3:**
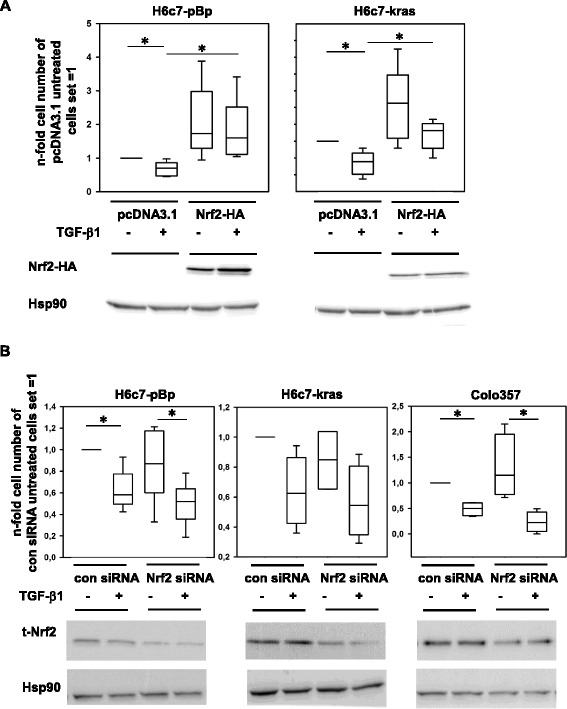
Nrf2 antagonizes the growth inhibiting effect of TGF-β1 on pancreatic ductal epithelial cells. **a** H6c7-pBp and H6c7-kras cells that were transfected with a control vector (pcDNA3.1) or Nrf2-HA and left untreated or were treated with 10 ng/ml TGF-β1 for 48 h. Determination of vital cell numbers by trypan blue exclusion and cell counting. Verification of Nrf2 overexpression was performed by western blotting using an HA-specific antibody and Hsp90 as loading control. **b** H6c7-pBp, H6c7-kras and Colo357 cells were transfected either with control siRNA or Nrf2 siRNA and left untreated or were treated with 10 ng/ml TGF-β1 for 48 h. Determination of vital cell numbers by trypan blue exclusion and cell counting. Verification of Nrf2 knockdown was performed by western blotting using a t-Nrf2 antibody and Hsp90 as loading control. Data of vital cell numbers are presented as Box-Plots of 4-6 independent experiments. * = *p* < 0.05

Next it was analyzed how Nrf2 impacts on cell growth of pancreatic ductal epithelial cells. For this purpose, H6c7-pBp and H6c7-kras cells exhibiting low and moderate basal Nrf2 expression, respectively (see Fig. 2), were transfected with Nrf2-HA and being either left untreated or treated with 10 ng/ml TGF-β1. After 48 h, vital cell numbers were determined. Compared to control transfected cells, in which TGF-β1 treatment significantly reduced the number of vital H6c7-pBp and H6c7-kras cells by 30 and 46.6 %, respectively, overexpression of Nrf2 clearly diminished the growth inhibiting effect of TGF-β1 treatment on both cell lines (10 and 31 %, respectively, compared to untreated cells) (Fig. 3a). The transfection efficiency was confirmed by detecting the HA-tag by western blotting using an HA-specific antibody for specific detection of recombinant Nrf2 (Fig. 3a bottom panel) and realtime PCR based determination of elevated expression of the Nrf2 target gene NQO1 (data not shown).

In order to verify the interference of Nrf2 with the growth inhibitory effect of TGF-β1 in all three pancreatic ductal epithelial cell lines, Nrf2 siRNA knockdown experiments were conducted. As demonstrated in Fig. 3b, knockdown of Nrf2 clearly enhanced the growth inhibiting effect of TGF-β1 in H6c7-pBp, H6c7-kras and Colo357 cells. Transfection efficiency was confirmed by detecting endogenous total-Nrf2 by western blotting (Fig. 3b, bottom panel) and realtime PCR based determination of reduced expression of the Nrf2 target gene NQO1 (data not shown). Overall, these data indicate that Nrf2 potently antagonizes the growth inhibiting function of TGF-β1 in human pancreatic ductal epithelial cells.

### Nrf2 increases proliferation of benign, premalignant and malignant pancreatic ductal epithelial cells

**Fig. 4:**
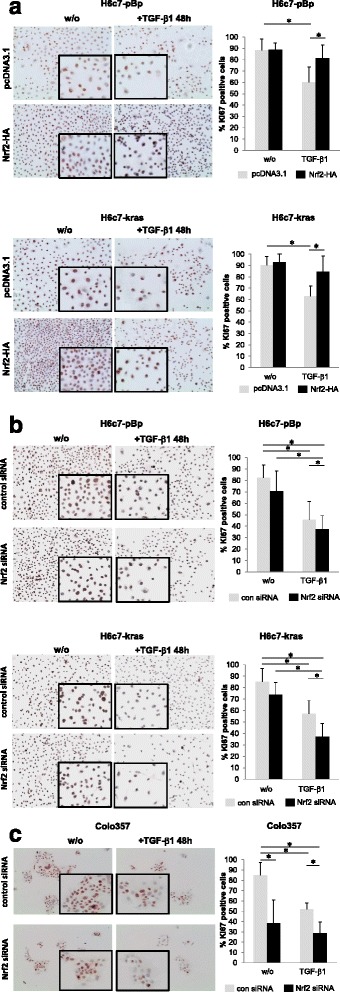
Nrf2 increases proliferation of benign, premalignant and malignant pancreatic ductal epithelial cells. Representative Ki67 stainings of (**a**) H6c7-pBp and H6c7-kras cells that were transfected with a control vector (pcDNA3.1) or Nrf2-HA and left untreated or were treated with 10 ng/ml TGF-β1 for 48 h, and (**b**) H6c7-pBp, H6c7-kras and (**c**) Colo357 cells that were transfected either with control siRNA or Nrf2 siRNA and left untreated or were treated with 10 ng/ml TGF-β1 for 48 h. Magnification x280 (magnification of images in frames x540). Quantification of Ki67 positive cells was performed at a 200-fold magnification by choosing 5 representative visual fields, in each counting positively stained as well as negative cells along a diagonal line using Microsoft Powerpoint 2007 in order to calculate the percentage of Ki67 positive cells. If less than ten cells touched the line, all captured cells of the visual field were counted. Data are expressed as mean ± SD of counted cells from 5 visual fields. * = *p* < 0.05

To elucidate how Nrf2 antagonizes the growth inhibiting effect of TGF-β1, proliferation of H6c7-pBp, H6c7-kras and Colo357 cells was analyzed in dependence on Nrf2 and TGF-β1. As demonstrated in Fig. 4a-c, TGF-β1 treatment drastically reduced the overall cell number as well as the number of Ki67 positive H6c7-pBp, H6c7-kras and Colo357 cells that had been transfected with the empty vector. In contrast, overexpression of Nrf2 clearly attenuated this effect of TGF-β1 and increased the cell density along with the number of Ki67 positive H6c7-pBp and H6c7-kras cells (Fig. 4a). In line with these findings, knockdown of Nrf2 in H6c7-pBp, H6c7-kras and Colo357 cells increased the antiproliferative effect of TGF-β1 as indicated by a further reduced cell number and less Ki67 expressing cells (Fig. 4b, c). Similar results were obtained by MTT assay (data not shown) supporting the view that Nrf2 antagonizes the growth inhibiting effect of TGF-β1 by counteracting its antiproliferative activity in pancreatic ductal epithelial cells.

### Nrf2 only slightly impact on basal and TGF-β1 induced apoptosis in pancreatic ductal epithelial cells

**Fig. 5:**
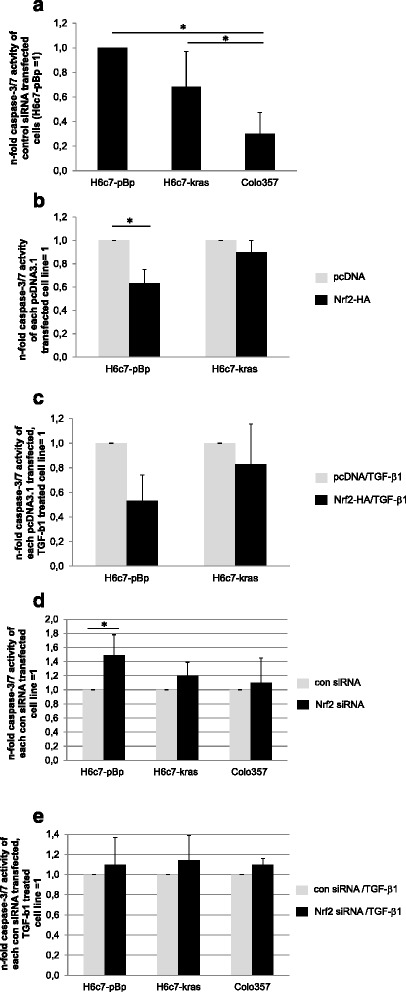
Nrf2 hardly impact on basal and TGF-β1 induced apoptosis in pancreatic ductal epithelial cells. **a**, **d**, **e** H6c7-pBp, H6c7-kras and Colo357 cells were transfected either with control siRNA or Nrf2 siRNA or (**b**, **c**) H6c7-pBp and H6c7-kras cells were transfected with a control vector (pcDNA3.1) or Nrf2-HA. Then, cells were either left untreated or were treated with 10 ng/ml TGF-β1 for 48 h. Caspase-3/7 activity was determined and normalized to the cell number. **a** Basal caspase-3/7 activity of control siRNA, untreated H6c7-pBp, H6c7-kras and Colo357 cells, expressed as n-fold caspase-3/7 activity of H6c7-pBp cells which were set as 1. **b** Caspase-3/7 activity in H6c7-pBp and H6c7-kras after overexpression of Nrf2-HA or (**d**) siRNA mediated Nrf2 suppression, expressed as n-fold caspase-3/7 activity of the corresponding control transfected cell line which was set as 1. **c** + **e** Caspase-3/7 activity in TGF-β1 treated H6c7-pBp and H6c7-kras after (**c**) overexpression of Nrf2-HA or (**e**) siRNA mediated Nrf2 suppression, expressed as n-fold caspase-3/7 activity of the corresponding control transfected, TGF-β1 treated cell line which was set as 1. Data are presented as mean ± SD of 3-5 independent experiments. * = *p* < 0.05

Since the growth inhibiting effect of TGF-β1 may also involve an increase of apoptosis, we next investigated the impact of Nrf2 on basal and TGF-β1 mediated apoptosis in the three pancreatic ductal epithelial cell lines. As shown in Fig. 5a, the basal apoptotic rate as determined by caspase-3/7 activity correlated well with the basal Nrf2 expression in the three pancreatic ductal epithelial cell lines. Hence, H6c7-pBp cells with the lowest basal Nrf2 activity (see also Fig. 2) exhibited the highest basal caspase-3/7 activity while Colo357 cells - showing the highest Nrf2 expression - were characterized by the lowest caspase-3/7 activity. Accordingly, when overexpressing Nrf2 in H6c7-pBp cells the basal (Fig. 5b) as well as the TGF-β1 mediated (Fig. 5c) apoptotic rate was clearly reduced, whereas Nrf2 overexpression in H6c7-kras cells only slightly affected basal and TGF-β1 dependent apoptosis. In line with the overexpression experiments, suppression of Nrf2 by siRNA transfection mainly increased the basal caspase-3/7 activity of H6c7-pBp cells but only slightly in H6c7-kras and Colo357 cells (Fig. 5d). Moreover, the TGF-β1 mediated apoptosis was marginally affected by Nrf2 suppression in all three cell lines (Fig. 5e). In order to validate these moderate Nrf2 mediated effects on apoptosis induction observed in H6c7-pBp and H6c7-kras cells by caspase-3/7 activity assay, PARP cleavage was analysed in the same cells by western blotting. Even though slight differences in the full length form of PARP were observed, cleaved PARP could be detected in neither sample (Additional file 2: Figure S1). These data indicate that the interference of Nrf2 with the growth suppression by TGF-β1 mainly relies on the reversal of its antiproliferative activity (see above) and to lesser extent on the protection from TGF-β1 dependent apoptosis.

### Nrf2 modulates MAPK and Smad signaling in benign, premalignant and malignant pancreatic ductal epithelial cells

**Fig. 6:**
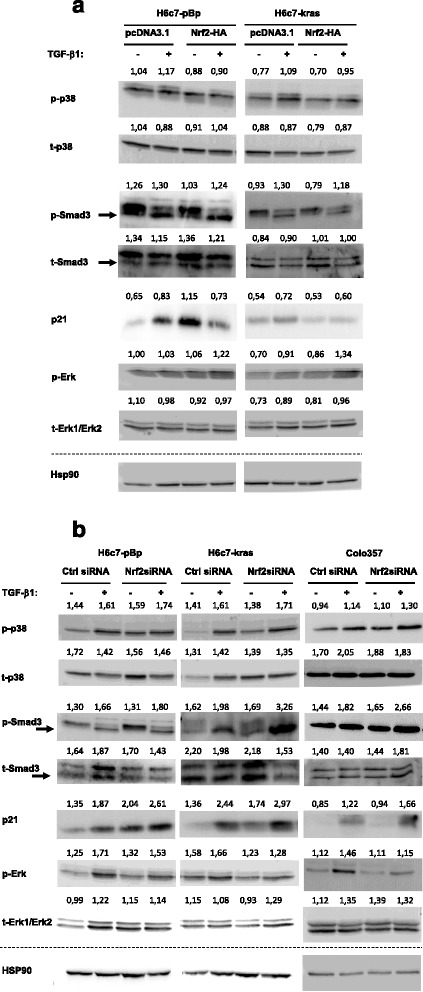
Nrf2 modulates MAPK and Smad signaling in benign, premalignant and malignant pancreatic ductal epithelial cells. **a** H6c7-pBp and H6c7-kras cells were transfected with a control vector (pcDNA3.1) or Nrf2-HA or (**b**) H6c7-pBp, H6c7-kras and Colo357 cells were transfected either with control siRNA or Nrf2 siRNA. Then, cells were either left untreated or were treated with 10 ng/ml TGF-β1 for 48 h. Representative western blots of 3-4 independent experiments showing expression of phosphorylated and total p38 (p-/t-p38), phosphorylated Smad3 (p-Smad3, marked by arrow) and total-Smad2/3 (t-Smad3 marked by arrow), phosphorylated and total Erk (p-/t-Erk) and p21. Hsp90 was detected as loading control. Numbers above each band indicate average band intensities determined by densitometry. Values of the proteins of interest were divided by the values of the corresponding loading control (Hsp90)

Next it was investigated how Nrf2 antagonizes TGF-β1 mediated growth inhibition. For this purpose the impact of Nrf2 on relevant signaling pathways, such as MAPK and Smad signaling as well as the expression of the cyclin-dependent kinase inhibitor 1 (p21) was analysed. As shown in Fig. 6a, overexpression of Nrf2 which was demonstrated by detecting the HA-tag of recombinant Nrf2 (see Fig. 3a) reduced basal as well as TGF-β1 mediated phosphorylation of p38. Likewise, Smad3 phosphorylation was also reduced by Nrf2 overexpression, albeit at lesser extent (Fig. 6a, Additional file 1: Figure S2A + B). Conversely, Nrf2 overexpression increased phosphorylation of the proliferation associated MAPK Erk in H6c7-pBp and H6c7-kras cells (Fig. 6a, Additional file 1: Figure S2A+B). Moreover, expression levels of p21 were reduced by Nrf2 overexpression in H6c7-kras cells in the absence and presence of TGF-β1 while in H6c7-pBp cells a marked effect of Nrf2 overexpression was observed solely in the presence of TGF-β1 (Fig. 6a, Additional file 1: Figure S2A + B).

To confirm the effects of Nrf2 on these growth controlling mediators, Nrf2 knock-down experiments were conducted with all three pancreatic ductal epithelial cell lines. siRNA mediated suppression of Nrf2 in H6c7-pBp, H6c7-kras and Colo357 cells, as demonstrated by detection of reduced levels of endogenous total-Nrf2 (see Fig. 3b), enhanced basal as well as TGF-β1 induced phosphorylation of p38 and Smad3 along with an elevated expression of p21. By contrast, TGF-β1 mediated phosphorylation of Erk was clearly reduced in the absence of Nrf2 (Fig. 6b, Additional file 1: Figure S2C + D). Overall, these data support the view that Nrf2 antagonizes TGF-β1 mediated growth suppression by attenuating the growth inhibiting (p38 and Smad3) signaling pathways and concomitantly enhancing growth promoting Erk-signaling.

## Discussion

The dual role of TGF-β1 in tumorigenesis of epithelial tumors is well established. Under physiological conditions, TGF-β1 is an essential cell growth inhibitor and thereby prevents epithelial hyperproliferation and the onset of epithelial tumors. However, TGF-β1 becomes a potent tumor promoter through its capacity to initiate EMT, to enhance cell migration and invasion and to favour chemoresistance [12, 13], applying also to PDAC [38–40]. Moreover, experimental evidence indicate that this functional switch might occur already at early stages in tumorigenesis when epithelial cells still do not harbour any of the driver mutations or at least only single mutations, such as in the *k-ras* oncogene [37, 39]. This functional switch of TGF-β1 leading to reprogramming of its activated signaling pathways seems to be context dependent, albeit the underlying mechanisms are still not fully understood. One mechanism explaining the TGF-β1 paradox might be the differential activation of MAPK such as Erk in benign and malignant epithelial cells [12]. Many tumors exhibit constitutive high Erk activation due to a mutation in the *ras* oncogene, leading to sustained TGF-β1 synthesis and signaling. In line with this, Gotzmann et al. showed that cooperation of TGF-β1 signaling and oncogenic expression of *Ha-ras* promotes a mesenchymal and invasive phenotype in hepatocytes [41]. Furthermore, sustained MAPK activation can also result from prolonged exposure to a plethora of inflammatory mediators (e.g. IL-6, TNF-α, TGF-β1) upon chronic inflammation [42, 43]. Thus, our study extends this view by demonstrating that sustained high expression and activity of the antioxidative transcription factor Nrf2 essentially contributes to deregulation of TGF-β1 signaling by reducing Smad3 and p38 activation and augmenting Erk activation resulting in reduced p21 expression and enhanced proliferation of pancreatic ductal epithelial cells. One can speculate that an elevated Nrf2 activity leads to changes in the assembly of TGF-β I (Alk1 and Alk5) and TGF-β II receptors, thereby favouring an altered recruitment of downstream mediators that can initiate Smad independent/non-canonical pathways like Ras-ERKs. Thus, further studies have to unravel in more detail how Nrf2 suppresses TGF-β1 mediated Smad3 and p38 activation and concomitantly enhance Erk signaling. Interestingly, we could observe these effects likewise in benign, premalignant and malignant pancreatic ductal epithelial cells suggesting that high Nrf2 activity can promote this functional switch of TGF-β1 already early in tumorigenesis. Favoring this hypothesis, we could detect enhanced expression of activated (phosphorylated) Nrf2 in nuclei of early PanINs being significantly associated with a higher proliferative activity exemplified by elevated Ki67 expression. Moreover, these data are in line with findings from an endogenous PDAC mouse model showing that oncogenic *kras* activation is one key mechanism leading to sustained Nrf2 expression and activity driving pancreatic tumorigenesis [25]. Hence, pancreata of Nrf2 deficient mice exhibited fewer PanINs with lower proliferative activity than the wildtype mice whereas no differences in the extent of apoptotic cells could be observed [25]. Thus, activation of the ras-raf-Erk pathway [25, 44] represents an important mechanism leading to enhanced Nrf2 expression and activity in tumor cells, applying also to pancreatic ductal epithelial cells. Accordingly, we could demonstrate higher basal Nrf2 expression and activity in H6c7-kras and Colo357 cells compared to benign H6c7-pBp cells. Important to note, constitutive Nrf2 activation is caused to a lesser extent by genetic alterations affecting either the Nrf2 inhibitor Keap1 or Nrf2 itself (accounting for 10–15 % in gallbladder and ovarian cancer or in lung cancer up to 30 % of enhanced Nrf2 activation) as well as epigenetic mechanisms accounting for enhanced Nrf2 activity in 20–30 % of tumors [45, 46]. Accordingly, we previously showed that PDAC cells with different Nrf2 activity exhibit similar Keap1 expression levels [18] supporting the view that sustained Nrf2 activation in PDAC cells is not caused by epigenetic or genetic alterations of Keap1 and rather than other factors. Besides the above mentioned oncogene induced Nrf2 activation, the exposure of epithelial cells to persistent metabolic and/or oxidative stress, e.g. caused by the presence of ROS releasing macrophages and neutrophils during the course of a chronic inflammation [47] is a major cause for constitutive elevated Nrf2 activity. Macrophages are not only abundant in chronic pancreatitis and PDAC [8] but start to accumulate around early PanINs. Hence, the growing inflammatory microenvironment together with oncogenic *kras* activation in ductal epithelial cells, which is present in almost all early PanINs [3], may promote upregulation of both Nrf2 and TGF-β1 activity already at this early stage of PDAC development. Supporting this view, we previously demonstrated that predominantly proinflammatory macrophages releasing ROS at high levels lead to nuclear accumulation of Nrf2 in colonic epithelial cells conferring apoptosis resistance towards TRAIL or the chemotherapeutic drug irinotecan [47]. This Nrf2 mediated apoptosis resistance relied on Nrf2 enhanced proteasomal gene expression [47], a mechanism that apparently also operates in PDAC cells [18].

In contrast to the findings of Lister et al., in our studies the effect of Nrf2 *per se* on cell growth of pancreatic ductal epithelial cells was minor [17] which might be explained by different cultivation periods after modulation of Nrf2 expression. Nrf2 essentially impacts on cell survival of pancreatic ductal epithelial cells by antagonizing the growth inhibitory effect of TGF-β1 by diminishing TGF-β1 induced p21 expression and accelerating Erk signaling resulting in increased proliferation. Besides a pro-proliferative function, Nrf2 has been described to confer profound protection from apoptosis by e.g. upregulation of anti-apoptotic proteins such as bcl-2 or bcl-xL [19, 20] or proteasomal genes such as s5a/psmd4 or α5/psma5 [18]. Thus, elevated Nrf2 expression is one pivotal mechanism underlying chemoresistance of many tumors, e.g. PDAC [18–21]. Accordingly, in this study the highest Nrf2 expression/activity could be correlated with the lowest apoptosis rate in Colo357 cells and *vice versa*. However, overexpression or knock down of Nrf2 in pancreatic ductal epithelial cells only slightly modulated TGF-β1 mediated apoptosis induction. Thus, Nrf2 apparently enhances the growth of pancreatic ductal epithelial cells predominantly by antagonizing the proliferation inhibiting effect of TGF-β1. Since this phenomenon could be observed not only in malignant but also in premalignant and benign epithelial cells, one can speculate that the switch of Nrf2 from tumor suppressor to a tumor promoter might occur quite early in tumorigenesis and in particular in the concomitant presence of TGF-β1.

## Conclusions

Overall this study provides a novel mechanism explaining the functional switch of Nrf2 and TGF-β1, both factors that exert anti-tumorigenic functions and contribute to cell/tissue homeostasis under physiological and acute inflammatory conditions but which likewise are able to promote tumor development. Hence, concomitant and persistent elevation of both factors leads to deregulation of the activated signaling pathways thereby fostering survival and growth of transformed cells as well as cell invasion [37]. Thus, this might also explain why therapeutic strategies targeting only either factor have been proven less effective. Therefore, future studies have to investigate whether concomitant targeting of Nrf2 and TGF-β1 will be more efficient in the treatment of highly malignant tumors such as PDAC.

## Additional files

Additional file 1: Figure S2. Nrf2 modulates MAPK and Smad signaling in benign, premalignant and malignant pancreatic ductal epithelial cells. A + B) H6c7-pBp and H6c7-kras cells were transfected with a control vector (pcDNA3.1) or Nrf2-HA or (C + D) H6c7-pBp, H6c7-kras and Colo357 cells were transfected either with control siRNA or Nrf2 siRNA. Then, cells were either left untreated or were treated with 10 ng/ml TGF-β1 for 48 h. A + C) Densitometric analysis of p-p38, p-Smad3, p-Erk and p21 expression normalised to Hsp90 expression in the indicated samples. Data are presented as mean ± SD of three independent experiments. B + D) Representative western blots of 3-4 independent experiments showing expression of phosphorylated and total p38 (p-/t-p38), phosphorylated Smad3 (p-Smad3 marked by arrow) and total-Smad3 (t-Smad3 marked by arrow) and phosphorylated and total Erk (p-/t-Erk). Hsp90 was detected as loading control. Numbers above each band indicate average band intensities determined by densitometry. Values of phosphorylated proteins were divided by the values of the corresponding total protein. Nrf2 overexpression was confirmed by detecting the HA-tag of recombinant Nrf2 (see Fig. 3a) and siRNA mediated suppression of endogenous Nrf2 was confirmed by detection of total-Nrf2 (t-Nrf2, see Fig. 3b). (PDF 273 kb) Additional file 2: Figure S1. Nrf2 hardly impact on basal and TGF-β1 induced apoptosis in pancreatic ductal epithelial cells. H6c7-pBp and H6c7-kras cells were transfected either with (A) a control vector (pcDNA3.1) or Nrf2-HA or (B) control siRNA or Nrf2 siRNA. Then, cells were either left untreated or were treated with 10 ng/ml TGF-β1 for 48 h. Cells were detached and one part was used for determining caspase-3/7 activity. The other part was used for cell counting and normalization of caspase-3/7 activity. Then, these cells were lysed in Laemmli buffer and analysed for PARP (full length 116 kD form and cleaved 89 kD form) by western blotting. Hsp90 was detected as loading control. One representative result is shown. (PDF 159 kb)

## Abbreviations

CP: chronic pancreatitis
HO-1: Hemoxygenase-1
MAPK: Mitogen-activated protein kinase
NQO1: NAD(P)H dehydrogenase [quinone] 1
Nrf2: Nuclear factor E2 related factor-2
PanIN: pancreatic intraepithelial neoplasia
PDAC: pancreatic ductal adenocarcinoma
TGF-β1: Transforming Growth Factor beta-1
