# Supplementary figures and images for: The anti-oxidative transcription factor Nuclear factor E2 related factor-2 (Nrf2) counteracts TGF-β1 mediated growth inhibition of pancreatic ductal epithelial cells -Nrf2 as determinant of pro-tumorigenic functions of TGF-β1
Source: BMC Cancer. 2016 Feb 25;16:155. doi: 10.1186/s12885-016-2191-7 (PMC4766703; doi:10.1186/s12885-016-2191-7)

A)

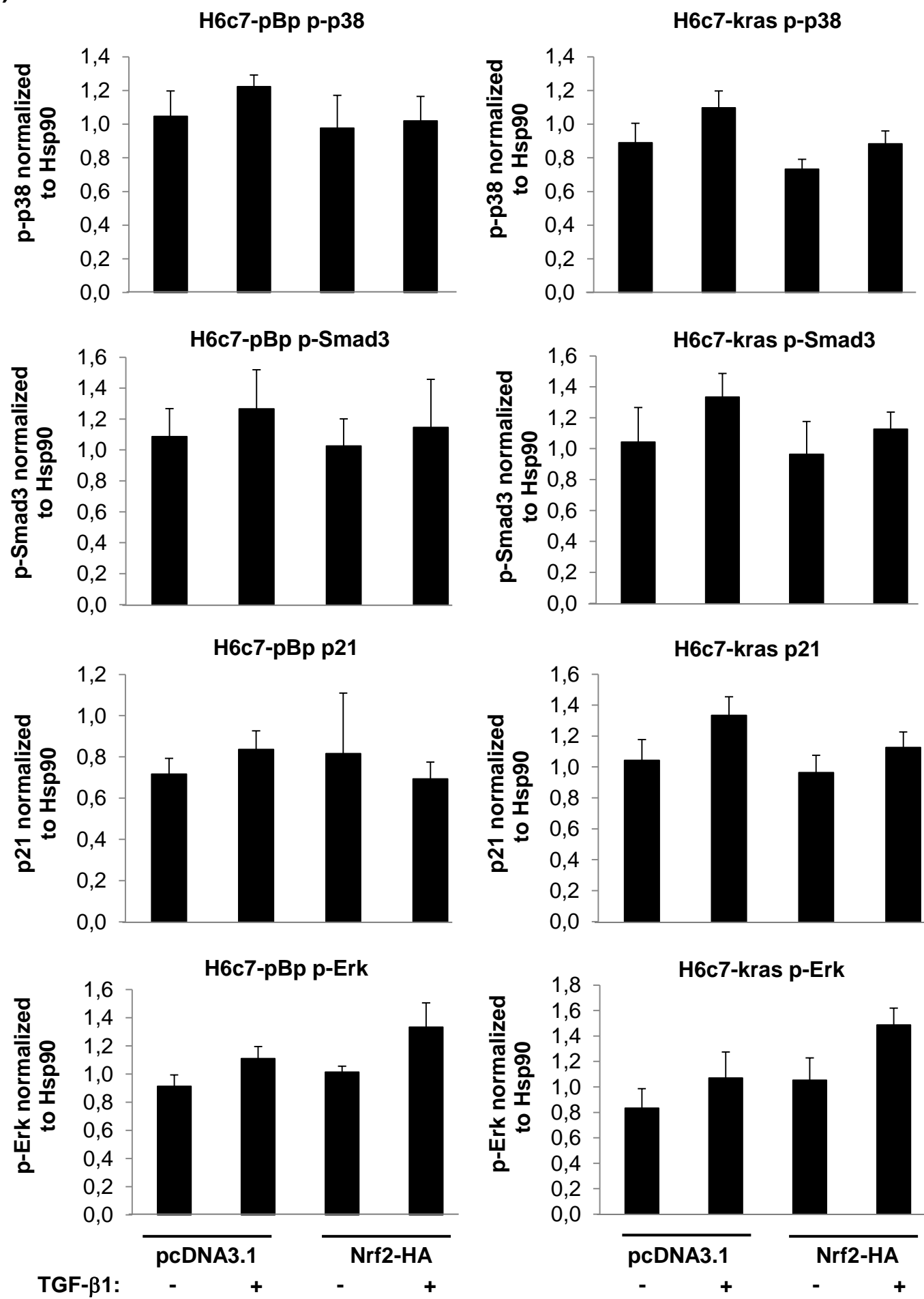

Supplementary Figure 2

B)

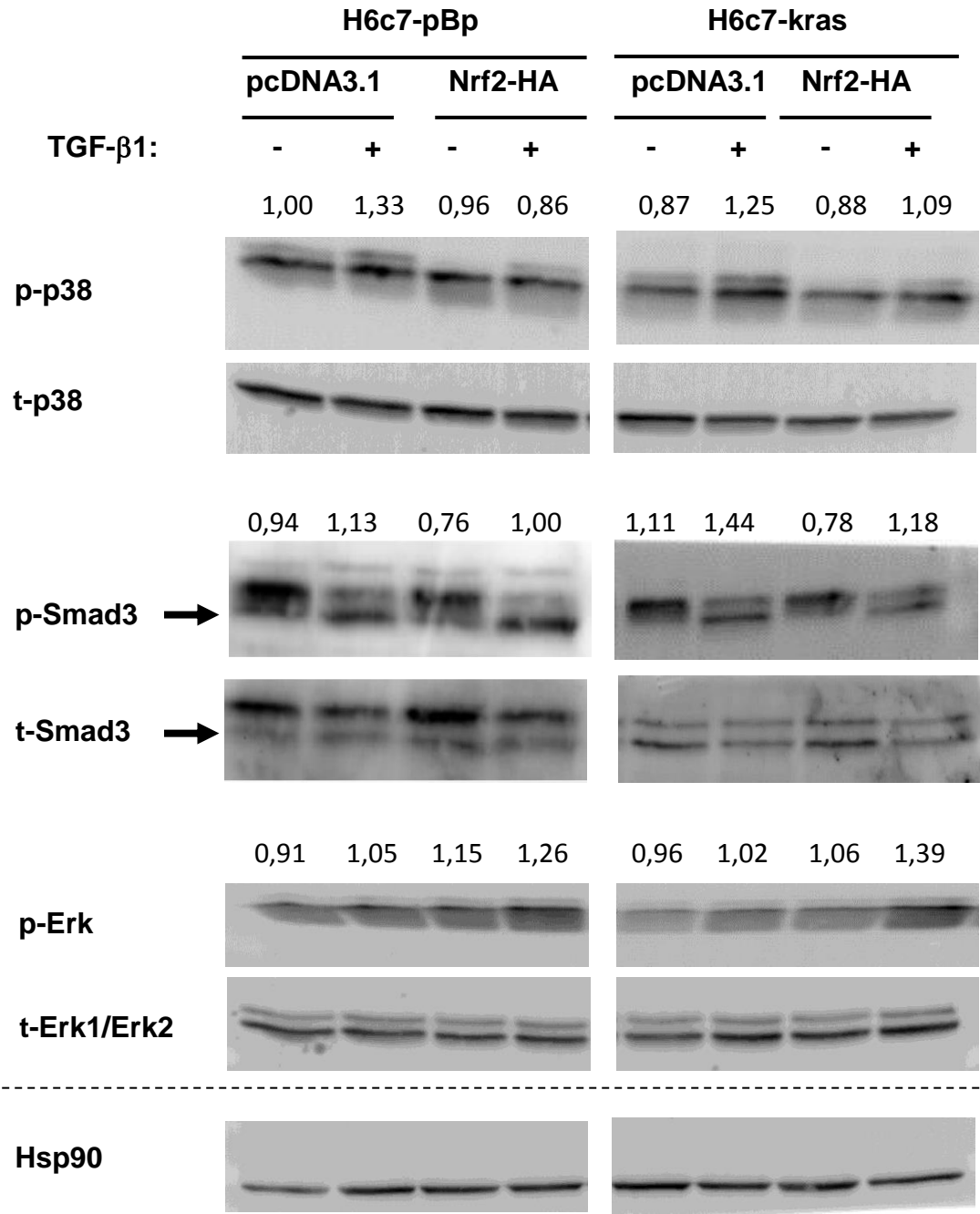

c)

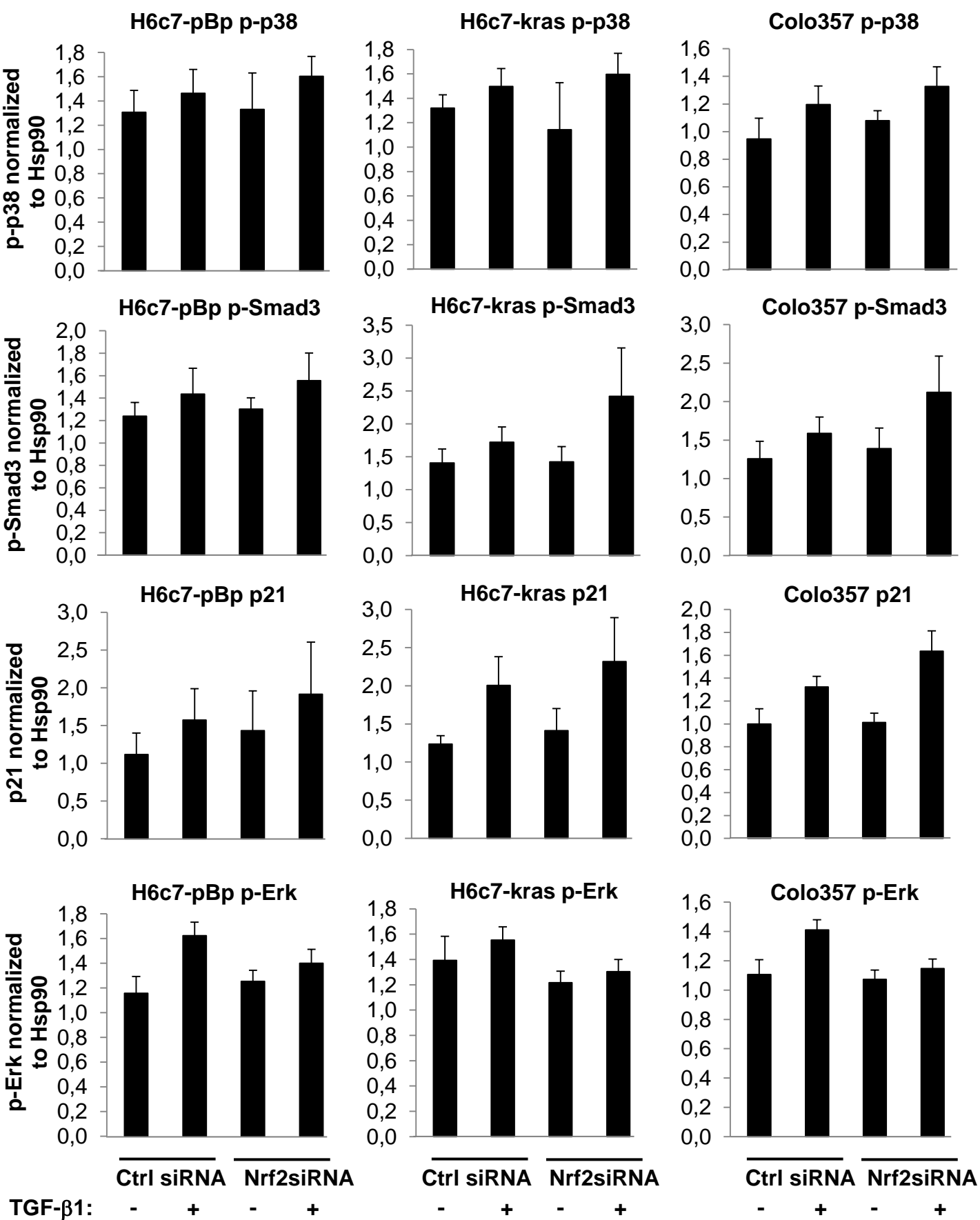

Supplementary Figure 2

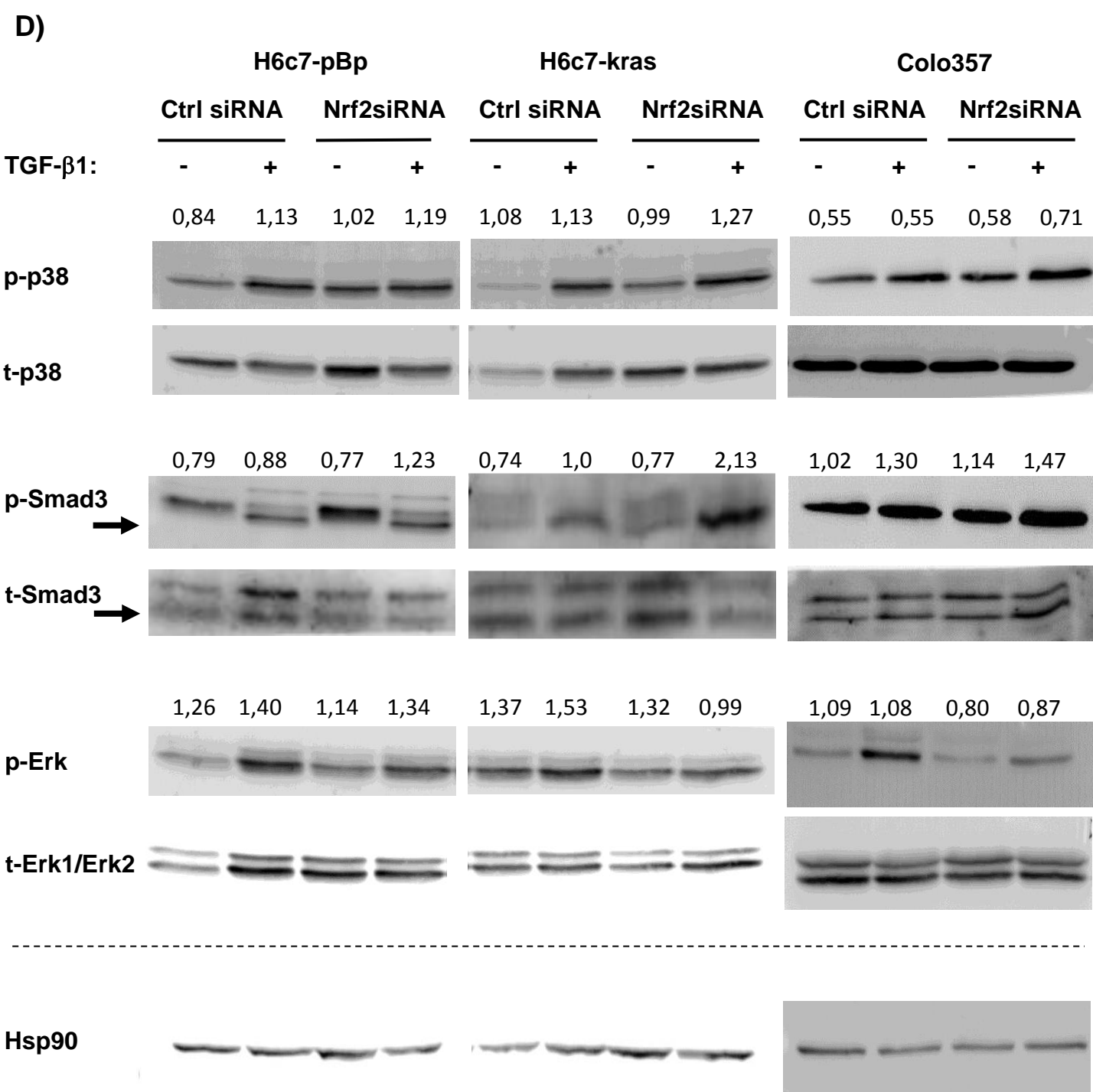

**Supplementary Figure 2**

Supplement: Additional file 1: Figure S2. — Nrf2 modulates MAPK and Smad signaling in benign, premalignant and malignant pancreatic ductal epithelial cells. A + B) H6c7-pBp and H6c7-kras cells were transfected with a control vector (pcDNA3.1) or Nrf2-HA or (C + D) H6c7-pBp, H6c7-kras and Colo357 cells were transfected either with control siRNA or Nrf2 siRNA. Then, cells were either left untreated or were treated with 10 ng/ml TGF-β1 for 48 h. A + C) Densitometric analysis of p-p38, p-Smad3, p-Erk and p21 expression normalised to Hsp90 expression in the indicated samples. Data are presented as mean ± SD of three independent experiments. B + D) Representative western blots of 3-4 independent experiments showing expression of phosphorylated and total p38 (p-/t-p38), phosphorylated Smad3 (p-Smad3 marked by arrow) and total-Smad3 (t-Smad3 marked by arrow) and phosphorylated and total Erk (p-/t-Erk). Hsp90 was detected as loading control. Numbers above each band indicate average band intensities determined by densitometry. Values of phosphorylated proteins were divided by the values of the corresponding total protein. Nrf2 overexpression was confirmed by detecting the HA-tag of recombinant Nrf2 (see Fig. 3a) and siRNA mediated suppression of endogenous Nrf2 was confirmed by detection of total-Nrf2 (t-Nrf2, see Fig. 3b). (PDF 273 kb) [file 12885_2016_2191_MOESM1_ESM.pdf]

A)

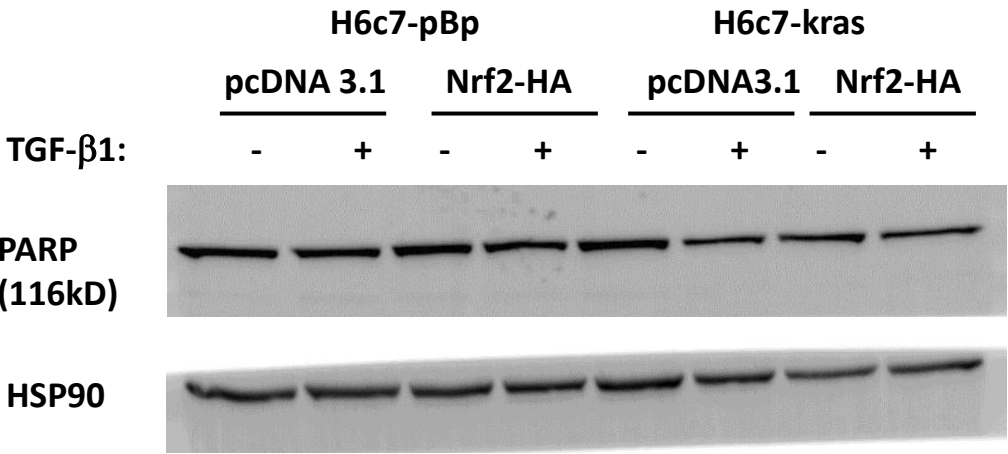

B)

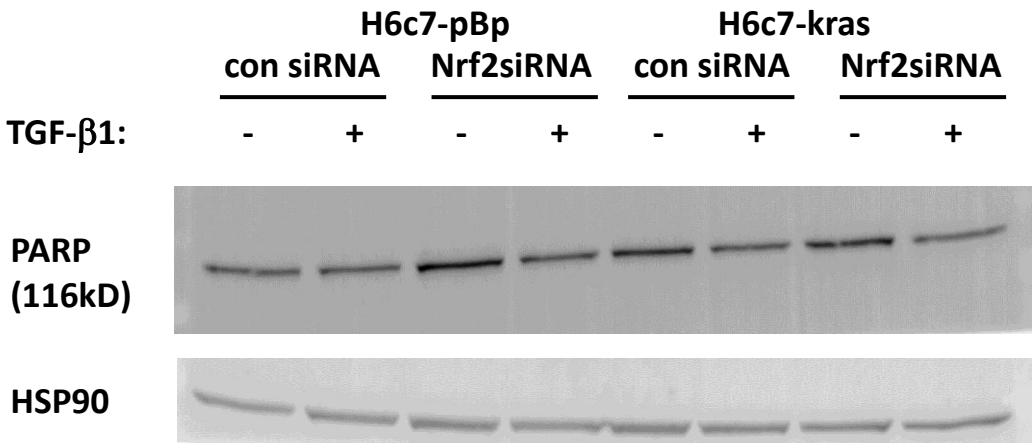

Supplement: Additional file 2: Figure S1. — Nrf2 hardly impact on basal and TGF-β1 induced apoptosis in pancreatic ductal epithelial cells. H6c7-pBp and H6c7-kras cells were transfected either with (A) a control vector (pcDNA3.1) or Nrf2-HA or (B) control siRNA or Nrf2 siRNA. Then, cells were either left untreated or were treated with 10 ng/ml TGF-β1 for 48 h. Cells were detached and one part was used for determining caspase-3/7 activity. The other part was used for cell counting and normalization of caspase-3/7 activity. Then, these cells were lysed in Laemmli buffer and analysed for PARP (full length 116 kD form and cleaved 89 kD form) by western blotting. Hsp90 was detected as loading control. One representative result is shown. (PDF 159 kb) [file 12885_2016_2191_MOESM2_ESM.pdf]
